# Minimizing the Strong Screening Effect of Polyhedral Oligomeric Silsesquioxane Nanoparticles in Hydrogen-Bonded Random Copolymers

**DOI:** 10.3390/polym10030303

**Published:** 2018-03-11

**Authors:** Wei-Cheng Chen, Ruey-Chorng Lin, Shih-Min Tseng, Shiao-Wei Kuo

**Affiliations:** 1 Department of Materials and Optoelectronic Science, National Sun Yat-Sen University, Kaohsiung 80424, Taiwan; chwei566@gmail.com (W.C.); rueyclin01@gmail.com (R.C.); anniezeng1111@gmail.com (S.M.); 2 Department of Medicinal and Applied Chemistry, Kaohsiung Medical University, Kaohsiung 80424, Taiwan

**Keywords:** hydrogen bonding, screening effect, POSS nanoparticles, copolymers

## Abstract

A series of poly(vinylphenol-*co*-methacryisobutyl polyhedral oligomeric silsesquioxane) (PVPh-*co*-PMAPOSS) random copolymers have been synthesized through free radical copolymerizations of acetoxystyrene with methacryisobutyl POSS monomer and subsequent hydrazine monohydrate-mediated hydrolysis of the acetoxyl units. These random copolymers were characterized using nuclear magnetic resonance spectroscopy (NMR), Fourier transform infrared spectroscopy (FTIR), gel permeation chromatography (GPC), and differential scanning calorimetry (DSC), which revealed that the POSS content in the random copolymers could be varied by changing the POSS monomer feed ratio by ^1^H NMR analyses. This molecular design approach allowed us to investigate the thermal properties and hydrogen bonding interactions of these PVPh-*co*-PMAPOSS random copolymers in comparison with those of PVPh/PMAPOSS blend systems. Hydrogen bonding interactions were absent in the PVPh/PMAPOSS blend system, because of a strong screening effect; in contrast, the PVPh-*co*-PMAPOSS random copolymers experienced enhanced intramolecular hydrogen bonding that minimized the strong screening effect of the POSS nanoparticles.

## 1. Introduction

**Scheme 1:**
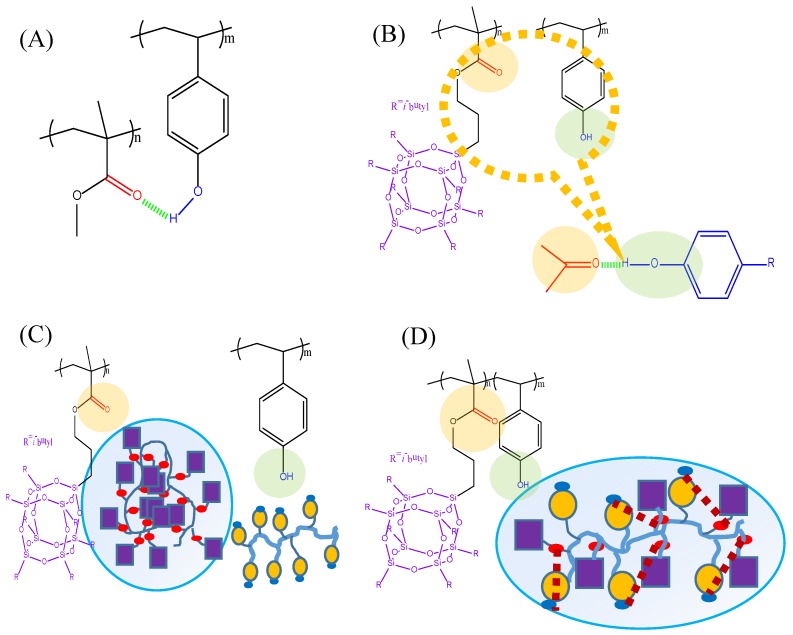
(**A**,**B**) Possible hydrogen bonding interactions in (**A**) polyvinylphenol/poly(methyl methacrylate) (PVPh/PMMA) and (**B**) poly(vinylphenol–methacryisobutyl polyhedral oligomeric silsesquioxane) (PVPh/PMAPOSS) blends; (**C**,**D**) Real hydrogen bonding interactions in (**C**) a PVPh/PMAPOSS blend and (**D**) a poly(vinylphenol–*co*–methacryisobutyl polyhedral oligomeric silsesquioxane) (PVPh-*co*-PMAPOSS) random copolymer.

Hydrogen bonding interactions in polymeric materials has garnered much interest over the past three decades for their ability to enhance thermal properties, improve miscibility behavior, induce self-assembly structures, and form nanocomposites [1,2,3,4,5,6,7]. The most widely investigated hydrogen-bonded polymer blend system is that of polyvinylphenol/poly(methyl methacrylate) (PVPh/PMMA), in which the OH units of PVPh interact with the C=O units of PMMA (Scheme 1A) [8,9,10,11,12,13,14]. Coleman et al. determined the miscibility behavior and inter-association equilibrium constant (*K*_A_ = 37.4) of such PVPh/PMMA blends [13]. They also proposed the concepts of functional group accessibility and intramolecular screening effects (γ) based on comparisons with the interactions of PVPh and ethyl isobutyrate (EIB) in solution and of analogous PVPh-*co*-PMMA random copolymers [15,16,17,18]. The intramolecular screening effect has been defined as the self-bending of a polymer chain as a result of same-chain contact, through long-range, but also local, connectivity effects. Furthermore, this group observed a high screening effect when PVPh was blended with a dendrimer-like polyester (γ = 0.8) [19], but a low effect with a linear PVPh/PMMA blend (γ = 0.3) [15,16,17,18,19].

In a previous study, we prepared, through atom transfer radical polymerization (ATRP), block copolymers featuring polyhedral oligomeric silsesquioxane (POSS) nanoparticles at the chain ends of PMMA (PMMA-*b*-POSS) and then blended them with phenolic and PVPh homopolymers [20]. The incorporation of POSS nanoparticles into polymeric materials appears promising because it can improve the thermal properties, lower the density, decrease the flammability, and enhance the self-assembly behavior of the polymers, all mediated by the functionality of the organic units in the POSS nanoparticles [21,22,23,24,25,26,27]. With the POSS nanoparticle positioned at the chain end, we observed a screening effect for PMMA-*b*-POSS blended with phenolic and PVPh homopolymers, with a value of γ of 0.65 [20]. Because of the limited content of POSS nanoparticles possible at the chain end of a PMMA homopolymer, we also examined the effects of grafting them onto the side chains of PMMA (PMA-POSS) through anionic polymerization [28]. Notably, hydrogen bonding interactions were lacking between the OH units of PVPh and the C=O units of PMA-POSS (Scheme 1B). This strong screening effect in PVPh/PMA-POSS blends resulted in immiscibility, with a value of γ equal to 1. Even when blended with the low-molecular-weight versions of phenolic resin and bisphenol A (BPA), no hydrogen bonding interactions occurred in the blend systems, presumably because strong aggregation of the POSS moieties on the PMMA side chains inhibited hydrogen bonding between the OH units of PVPh and the phenolic resin or BPA (Scheme 1C) [28].

Random copolymers usually feature a higher fraction of hydrogen bonding interactions and higher glass transition temperatures when compared with their corresponding polymer blend systems, due to compositional heterogeneities [14,18,29,30,31]. Thus, in this study, we attempted to induce hydrogen bonding between the C=O units of PMA-POSS and the OH units of PVPh through the free radical copolymerizations giving a series of PVPh-*co*-PMAPOSS random copolymers (Scheme 1D). This molecular design approach allowed us to investigate the thermal properties and hydrogen bonding interactions of these PVPh-*co*-PMAPOSS random copolymers and compare them with those of PVPh/PMAPOSS blend systems.

## 2. Materials and Methods 

### 2.1. Materials

The methacryisobutyl POSS (MA-POSS, C_35_H_74_O_14_Si_8_) was purchased from Hybrid Plastics (Hattiesburg, MS, USA). 4-Acetoxystryene was distilled from CaH_2_ under reduced pressure, and high-purity azobisisobutyronitrile (AIBN) was kept in a dry box; both were purchased from Alfa-Aesar (Ward Hill, MA, USA). All other chemicals were purchased from Aldrich (St. Louis, MO, USA) and used without further purification.

### 2.2. Polymerization

**Scheme 2:**
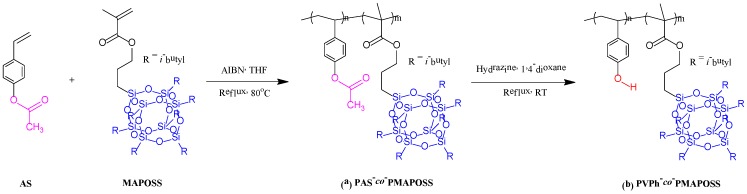
Synthesis of (**a**) poly(acetoxystyrene-*co*-methacryisobutyl POSS) (PAS-*co*-PMAPOSS) and (**b**) PVPh-*co*-PMAPOSS random copolymers.

All random copolymerizations were performed in a vacuum-line apparatus under an N_2_ atmosphere. Poly(acetoxystyrene-*co*-methacryisobutyl POSS) (PAS-*co*-PMAPOSS) copolymers were synthesized through free radical copolymerizations, using AIBN as the initiator, in dry tetrahydrofuran (THF) at 80 °C for 24 h. The reaction mixtures were poured into excess cold MeOH and agitated vigorously to precipitate the random copolymers. The PAS-*co*-PMAPOSS random copolymers were purified three times from THF/MeOH and were then dried in a vacuum oven at 80 °C to remove any residual solvent. The PVPh-*co*-PMAPOSS copolymers were prepared through hydrazine monohydrate-mediated hydrolysis of the PAS-*co*-PMAPOSS copolymers in 1,4-dioxane at room temperature for 2 days. The reaction products in THF were precipitated in deionized H_2_O. Concentration of the organic phase under vacuum distillation provided the PVPh-*co*-PMAPOSS copolymers as solids that were dried under vacuum. Scheme 2 summarizes the syntheses of the PAS-*co*-PMAPOSS and PVPh-*co*-PMAPOSS copolymers.

### 2.3. Characterization

Nuclear magnetic resonance (NMR) spectra were recorded using a Bruker ARX500 spectrometer (McKinley Scientific, Sparta, NJ, USA) with CDCl_3_ as the solvent and tetramethylsilane (TMS) as the external standard. The molecular weights of the random copolymers were measured through gel permeation chromatography (GPC) using a Waters 510 HPLC (GPC, Waters, Taipei, Taiwan) and THF as the eluent. Fourier transform infrared (FTIR) spectra were recorded at a spectral resolution of 4 cm^−1^ from 4000 to 400 cm^−1^ using a Bruker Tensor 27 FTIR spectrophotometer (Billerica, MA, USA) operated under an N_2_ atmosphere at room temperature, using the KBr disk method or film samples. Thermal properties were determined using a TA Q-20 differential scanning calorimeter (TA Instrument, New Castle, DE, USA); the measurement was made using a ca. 7 mg sample on the DSC sample cell and each sample was cooled quickly to room temperature from the melt of the first heating scan and then scanned from 25 to 200 °C at a heating rate of 20 °C·min^−1^. Wide-angle X-ray diffraction (WAXD) analyses of the random copolymers were performed using the BL17A1 wiggler beamline of National Synchrotron Radiation Research Center (NSRRC) of Hsinchu,, Taiwan, with a wavelength of 1.32 Å. Samples of the random copolymers were sealed between two Kapton windows. Temperature-resolved measurements were taken at various temperatures on a heating stage under an N_2_ atmosphere.

## 3. Results and Discussion

### 3.1. Synthesis of PAS-co-PMAPOSS Random Copolymers 

**Figure 1:**
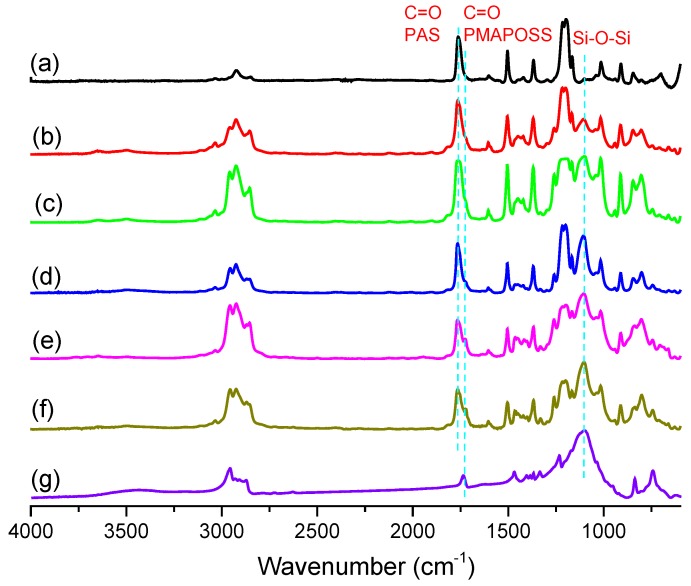
FTIR spectra, recorded at room temperature of (**a**) pure PAS; (**b**) PAS90-co-PMAPOSS10; (**c**) PAS80-co-PMAPOSS20; (**d**) PAS70-co-PMAPOSS30; (**e**) PAS60-co-PMAPOSS40; (**f**) PAS50-co-PMAPOSS50; and (**g**) pure PMAPOSS.

**Figure 2:**
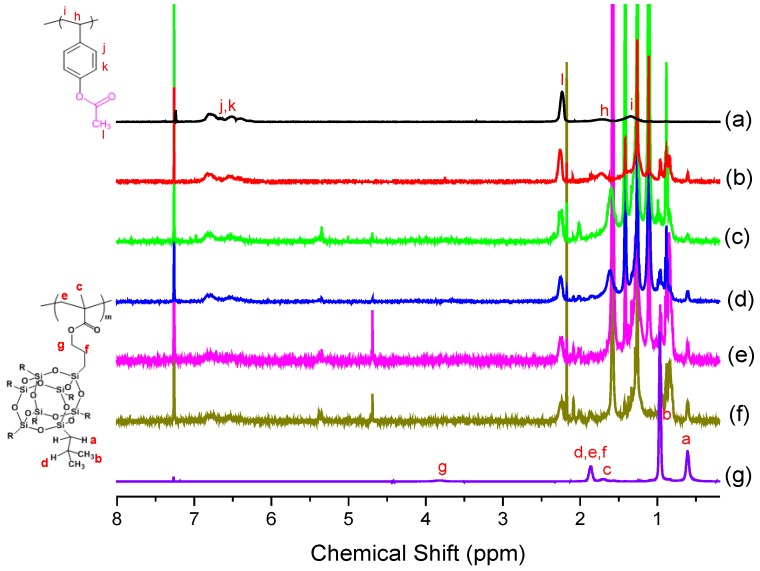
^1^H NMR spectra, recorded at room temperature, of (**a**) pure PAS, (**b**) PAS90-*co*-PMAPOSS10, (**c**) PAS80-*co*-PMAPOSS20, (**d**) PAS70-*co*-PMAPOSS30, (**e**) PAS60-*co*-PMAPOSS40, (**f**) PAS50-*co*-PMAPOSS50, and (**g**) pure PMAPOSS.

**Figure 3:**
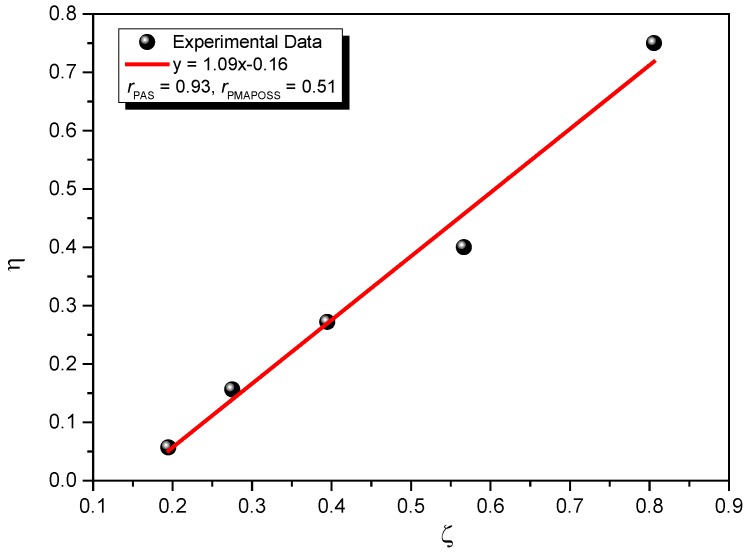
Kelen–Tudos plot of the PAS-*co*-PMAPOSS random copolymers.

Figure 1 displays the FTIR spectra, recorded at room temperature, of pure PAS, pure PMAPOSS, and various PAS-*co*-PMAPOSS random copolymers. The spectrum of pure PAS exhibits a strong characteristic C=O absorption peak at 1763 cm^–1^; for the pure PMAPOSS, two strong characteristic absorption peaks appeared at 1734 and 1096 cm^–1^, corresponding to the C=O and Si–O–Si stretching vibrations, respectively. The spectra of the PAS-*co*-PMAPOSS random copolymers featured all three of these characteristic peaks, with the intensities of those at 1734 and 1096 cm^–1^ increasing as the PMAPOSS content increased. All of these PAS-*co*-PMAPOSS copolymers were soluble in most common solvents. Figure 2 displays ^1^H NMR spectra of the pure PAS, pure PMAPOSS, and various PAS-*co*-PMAPOSS random copolymers, measured in CDCl_3_ as the solvent. The spectrum of pure PAS featured a signal for the CH_3_ units (*l*) at 2.25 ppm, with a broad resonance from 6.20 to 6.94 ppm corresponding to the aromatic protons (*j* and *k*); the spectrum of the pure PMAPOSS featured signals for the isobutyl groups at 0.60 (*a*), 0.97 (*b*), and 1.87 (*d*) ppm, with an integration ratio of 2:6:1, as well as a signal for the side-chain OCH_2_ (*g*) units at 3.80 ppm. The peaks (*e*) and (*f*) are overlapped with peak (*d*) for pure PMAPOSS. For all of the PAS-*co*-PMAPOSS random copolymers, the spectra revealed signals for both the aromatic protons of the PAS segment and the isobutyl protons (peak (*a*)), consistent with the copolymerization of PMAPOSS with the AS monomer. Based on integration of these two characteristic peaks, we calculated the mole percentages of the PAS and PMAPOSS segments in each random copolymer, using the following equation: PMAPOSS (%) = *A*_0.60_/2/(*A*_0.60_/2 + *A*_6.90_/4), where *A*_0.60_ is the integrated area of the signal for the isobutyl proton (peak (*a*)) of PMAPOSS, and *A*_6.90_ is the integrated area of the signals for the aromatic protons of the PAS segment. Table 1 lists the feed ratios of the AS and MAPOSS monomers and the resulting PMAPOSS contents in the PAS-*co*-PMAPOSS random copolymers, as determined from the ^1^H NMR spectroscopic analyses. We determined the molecular weights through GPC analyses. In addition, we calculated the reactivity ratios (*r*_PAS_ and *r*_PMAPOSS_) using the Kelen and Tudos method [14,31,32]; Figure 3 displays the results graphically. We obtained values of *r*_PAS_ and *r*_PMAPOSS_ of 0.93 and 0.51, respectively, indicating a tendency for random copolymerization of these two monomers.

**Figure 4:**
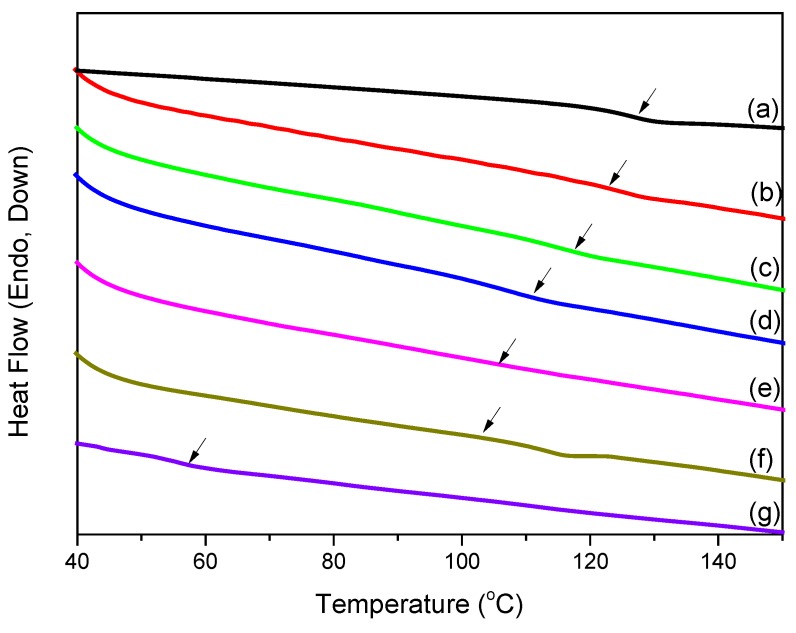
DSC thermograms of (**a**) pure PAS; (**b**) PAS90-*co*-PMAPOSS10; (**c**) PAS80-*co*-PMAPOSS20; (**d**) PAS70-*co*-PMAPOSS30; (**e**) PAS60-*co*-PMAPOSS40; (**f**) PAS50-*co*-PMAPOSS50; and (**g**) pure PMAPOSS.

Figure 4 presents DSC thermograms of the pure PAS, pure PMAPOSS, and various PAS-*co*-PMAPOSS random copolymers, measured at temperatures ranging from 40 to 150 °C. The pure PAS and pure PMAPOSS provided values of *T*_g_ of approximately 127 and 56 °C, respectively; each PAS-*co*-PMAPOSS random copolymer also exhibited a single value of *T*_g_, in the range from 105 to 122 °C [33], suggesting that no macrophase separation occurred in these random copolymers.

### 3.2. Synthesis of PVPh-co-PMAPOSS Random Copolymers

**Figure 5:**
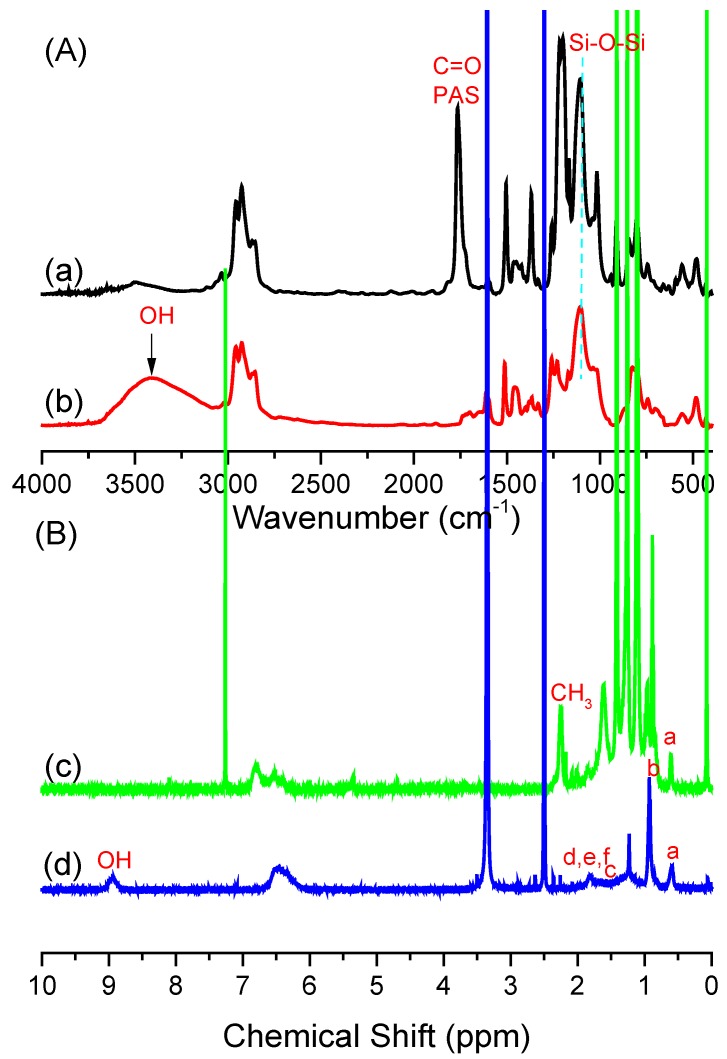
(**A**) FTIR spectra, recorded at room temperature, of (a) PAS60-*co*-PMAPOSS40, and (b) PVPh60-*co*-PMAPOSS40, and (**B**) ^1^H NMR spectra of (c) PAS60-*co*-PMAPOSS40 and (d) PVPh60-*co*-PMAPOSS40.

Figure 5 presents the FTIR and ^1^H NMR spectra of the PAS-*co*-PMAPOSS copolymers and after the selective hydrolysis of their acetoxyl groups to form PVPh-*co*-PMAPOSS random copolymers. The signal for C=O stretching at 1763 cm^–1^ (Figure 5a) disappeared after hydrolysis of the AS units of the PAS segments, with new OH absorption bands appearing at 3600–3200 cm^–1^ for the PVPh-*co*-PMAPOSS random copolymers (Figure 5b). Furthermore, the signal for the CH_3_ units at 2.25 ppm disappeared from the NMR spectra after hydrazinolysis with N_2_H_4_, and a new broad signal appeared for the phenolic OH groups at 8.93 ppm, consistent with high-yield syntheses of the PVPh-*co*-PMAPOSS random copolymers. In addition, the signals of the isobutyl units of the PMAPOSS segments remained after hydrazinolysis, confirming that the PMAPOSS and PAS segments were both incorporated within the PVPh-*co*-PMAPOSS random copolymers.

**Figure 6:**
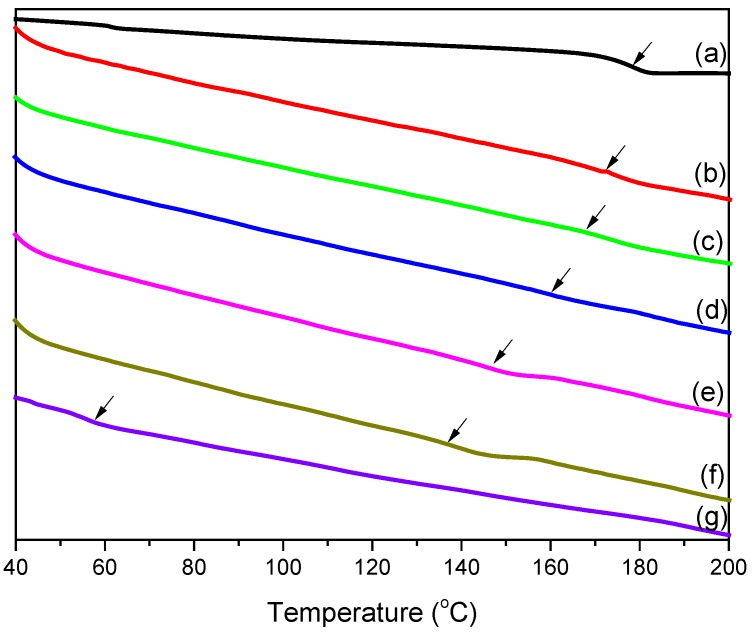
DSC thermograms of (**a**) pure PVPh, (**b**) PVPh90-*co*-PMAPOSS10, (**c**) PVPh80-*co*-PMAPOSS20, (**d**) PVPh70-*co*-PMAPOSS30, (**e**) PVPh60-*co*-PMAPOSS40, (**f**) PVPh50-*co*-PMAPOSS50, and (**g**) pure PMAPOSS.

**Figure 7:**
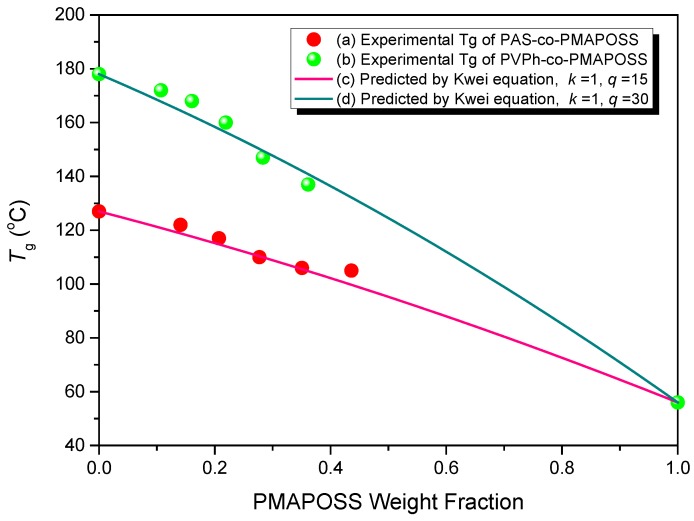
Experimental glass transition temperatures of (**a**) PAS-*co*-PMAPOSS and (**b**) PVPh-*co*-PMAPOSS random copolymers, and those predicted using the Kwei equation.

Figure 6 displays DSC thermograms of the pure PVPh, pure PMAPOSS, and various PVPh-*co*-PMAPOSS random copolymers, measured at temperatures ranging from 40 to 200 °C. The pure PVPh and pure PMAPOSS had values of *T*_g_ of approximately 178 and 56 °C, respectively. All of the PVPh-*co*-PMAPOSS random copolymers had values of *T*_g_ higher than those of the corresponding PAS-*co*-PMAPOSS random copolymers, suggesting that hydrogen bonding might have been occurring in the former. Figure 7 summarizes the glass transition temperature behavior of the PAS-*co*-PMAPOSS and PVPh-*co*-PMAPOSS random copolymers. The values of *T*_g_ for the PAS-co-PMAPOSS (Figure 7a) and PVPh-*co*-PMAPOSS (Figure 7b) random copolymers both exhibited positive deviations when compared with the Fox or linear rule; therefore, we used the Kwei equation [34] to predict the behavior of the glass transition temperatures of these two random copolymers, as follows:

(1) $$T_{g}=\frac{W_{1}T_{g1}+kW_{2}T_{g2}}{W_{1}+kW_{2}}+qW_{1}W_{2}$$

where *T*_gi_ represents the glass transition temperature of each copolymer segment (PAS, PVPh, or PMAPOSS), *W*_i_ is the weight fraction of each copolymer segment, and *k* and *q* are fitting constants.

We determined values of *k* and *q* of 1 and 15, respectively, for the PAS-*co*-PMAPOSS random copolymers (Figure 7c), and 1 and 30, respectively, for the PVPh-*co*-PMAPOSS random copolymers (Figure 7d). The larger positive value of *q* for the PVPh-*co*-PMAPOSS random copolymers indicates that their hydrogen bonding interactions were stronger than the dipole–dipole interactions of the PAS-*co*-PMAPOSS random copolymers.

### 3.3. Interaction and Phase Behavior of PVPh-co-PMAPOSS Random Copolymers

**Figure 8:**
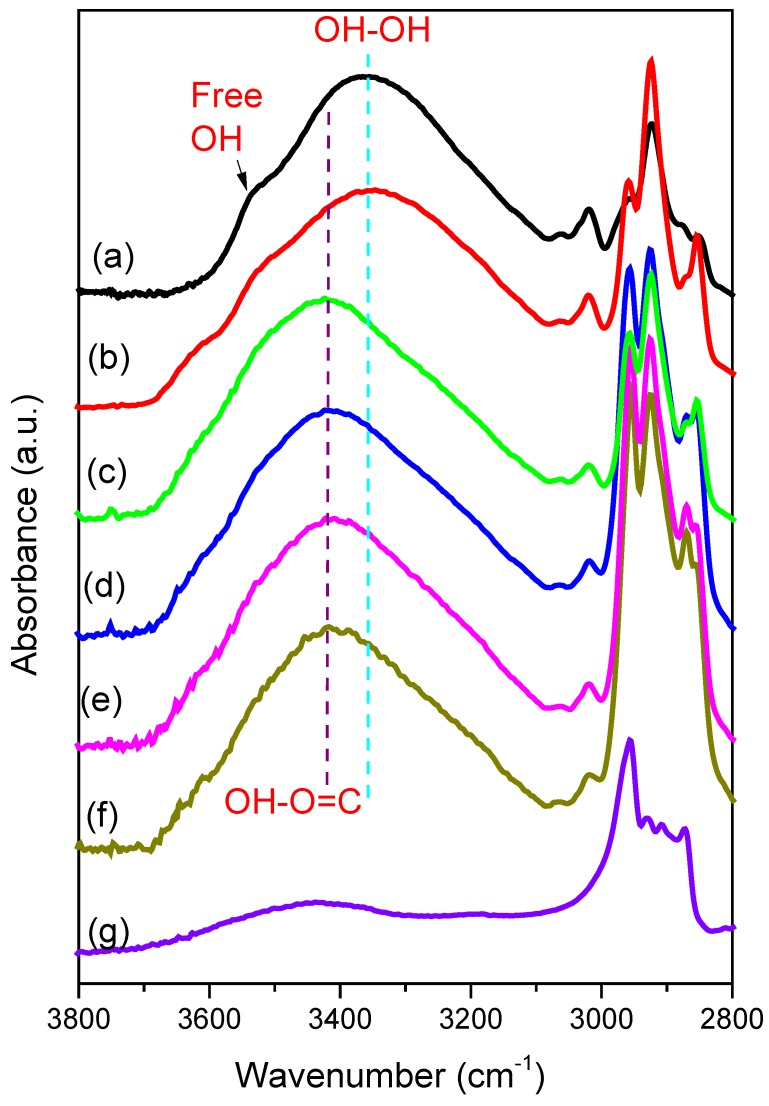
FTIR spectra (OH stretching region), recorded at room temperature, of (**a**) pure PVPh, (**b**) PVPh90-*co*-PMAPOSS10, (**c**) PVPh80-*co*-PMAPOSS20, (**d**) PVPh70-*co*-PMAPOSS30, (**e**) PVPh60-*co*-PMAPOSS40, (**f**) PVPh50-*co*-PMAPOSS50, and (**g**) pure PMAPOSS.

We used FTIR spectroscopy to characterize the hydrogen bonding interactions, both quantitatively for C=O stretching and qualitatively for OH stretching. Figure 8 presents FTIR spectra, in the region of the signals for OH stretching, of the PVPh-*co*-PMAPOSS random copolymers, measured at room temperature. The FTIR spectrum of the pure PVPh featured two distinct bands for OH stretching vibrations: a sharp band at 3525 cm^–1^ corresponding to the free OH units and a very broad band centered at 3360 cm^–1^ representing self-association hydrogen-bonded OH groups. The intensity of the signal for the free OH units decreased and the signal for the self-association hydrogen-bonded OH groups shifted to a higher wavenumber (to 3420 cm^–1^) upon as the content of PMAPOSS in the PVPh-*co*-PMAPOSS copolymer was increased. These changes are consistent with self-association OH⋯OH hydrogen bonds transferring to inter-association OH⋯O=C hydrogen bonds [14,18].

**Figure 9:**
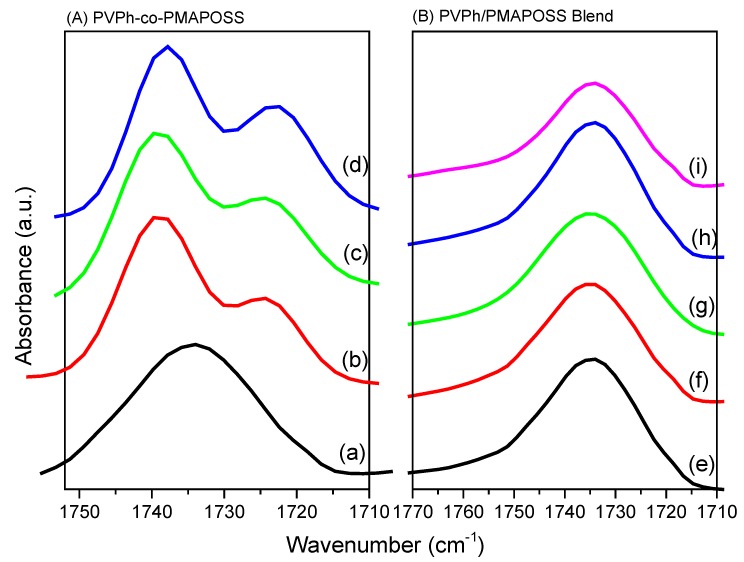
FTIR spectra (C=O stretching region), recorded at room temperature, of **(A)** PVPh-*co*-PMAPOSS random copolymers (**a**) pure PMAPOSS, (**b**) PVPh50-*co*-PMAPOSS50, (**c**) PVPh60-*co*-PMAPOSS40, and (**d**) PVPh70-*co*-PMAPOSS30] and **(B)** PVPh/PMAPOSS blends [(**e**) 0/100, (**f**) 20/80, (**g**) 40/60, (**h**) 60/40, and (**i**) 80/20.

We also used the signals for C=O stretching to investigate the formation of hydrogen bonds. Figure 9 displays FTIR spectra (C=O stretching region) for the PVPh-*co*-PMAPOSS random copolymers and PVPh/PMAPOSS blends at various PVPh contents. The signal for C=O stretching was split into two bands for each of the PVPh-*co*-PMAPOSS random copolymers. Figure 9a reveals a signal for the free C=O groups near 1734 cm^–1^ and a signal for the hydrogen-bonded C=O groups at 1722 cm^–1^. These two peaks could be fitted well by Gaussian functions. Using the appropriate absorptivity ratio (*a*_R_ = *a*_HB_/*a*_f_ =1.5), we observed that the fraction of hydrogen-bonded C=O groups increased as the PVPh content in the PVPh-*co*-PMAPOSS random copolymers was increased. In contrast, the C=O stretching region in the FTIR spectra of the PVPh/PMAPOSS blends recorded at room temperature exhibited (Figure 9b) [28] no signals for any hydrogen-bonded C=O units.

**Figure 10:**
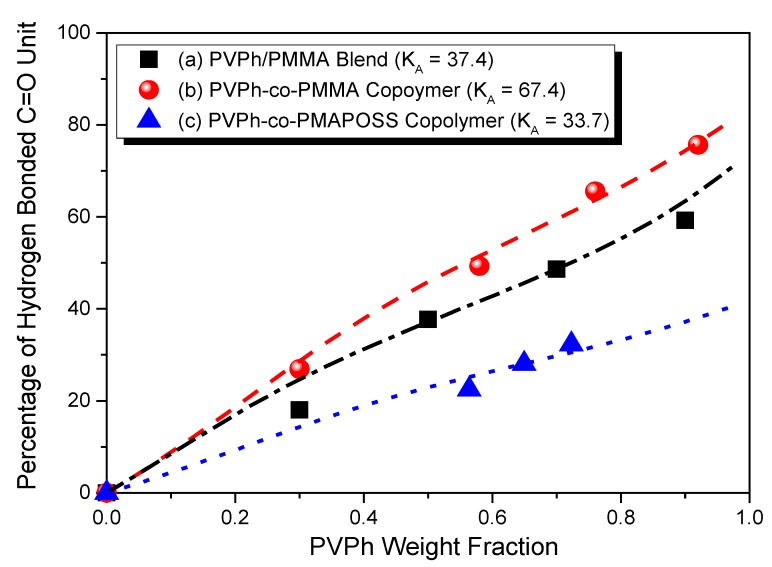
Percentages of hydrogen-bonded C=O units in (**a**) PVPh/PMMA blends, (**b**) PVPh-*co*-PMMA random copolymers, and (**c**) PVPh-*co*-PMAPOSS random copolymers, and those predicted using the Painter–Coleman association model to calculate values of *K*_A_.

Figure 10 summarizes the fractions of hydrogen-bonded C=O units of the PVPh-*co*-PMMA random copolymers, the PVPh/PMMA blends, and the PVPh-*co*-PMAPOSS random copolymers at room temperature, along with those predicted using the Painter–Coleman association model. As mentioned previously, the inter-association equilibrium constants (*K*_A_) were 37.4 for the PVPh/PMMA blends [13,14] and 67.4 for the PVPh-*co*-PMMA random copolymers [18]. We calculated a value of *K*_A_ of 33.7 for the PVPh-*co*-PMAPOSS random copolymers in this study. Thus, the intramolecular screening effect of the PVPh-*co*-PMAPOSS random copolymers yielded a value of γ of 0.5 (i.e., 33.7/67.4 = 0.5) when compared with the PVPh-*co*-PMMA random copolymers. Because intermolecular hydrogen bonding was absent in the PVPh/PMAPOSS blends, we suspect that hydrogen bonding occurred only through intramolecular or intra-chain interactions in the PVPh-*co*-PMAPOSS random copolymers, as displayed in Scheme 1D. As a result, we conclude that hydrogen bonding of the C=O units of PMA-POSS with the OH units of PVPh is possible in copolymers prepared through random copolymerization.

**Figure 11:**
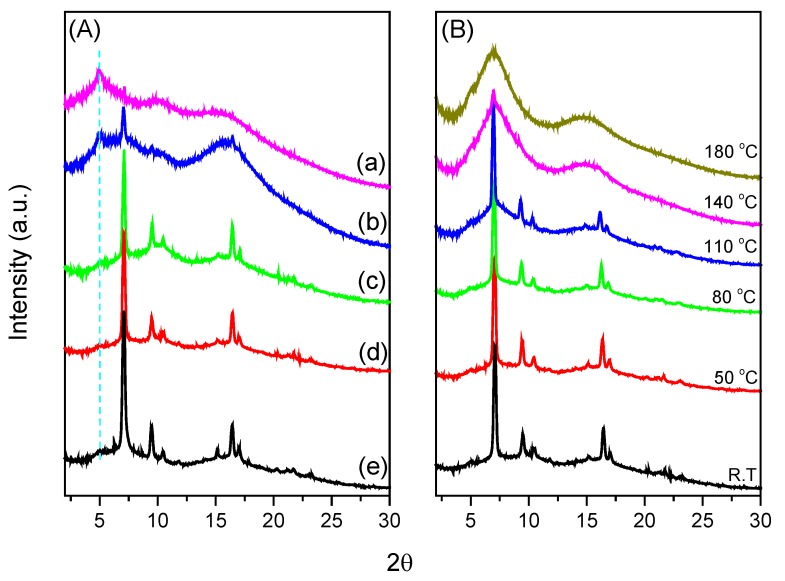
XRD patterns of (**A**) various PVPh-*co*-PMAPOSS random copolymers (**a**) PVPh90-*co*-PMAPOSS10, (**b**) PVPh80-*co*-PMAPOSS20, (**c**) PVPh70-*co*-PMAPOSS30, (**d**) PVPh60-*co*-PMAPOSS40, and (**e**) PVPh50-*co*-PMAPOSS50 and (**B**) the PVPh60-*co*-PMAPOSS40 random copolymer recorded at various temperatures.

Figure 11 displays the X-ray diffraction (XRD) patterns of various PVPh-*co*-PMAPOSS random copolymers and of the PVPh60-*co*-PMAPOSS40 random copolymer, recorded at various temperatures. Figure 11A reveals that the four main diffraction peaks at 7.06°, 9.49°, 10.48°, and 16.40° become stronger as the PMAPOSS content in the random copolymers increased; these signals correspond to *d*-spacings of 10.8, 8.0, 7.3, and 4.7 Å, respectively, similar to those found in the crystalline structure of POSS nanoparticles [35]. We also observed a diffraction peak at a value of 2θ of 4.96°, corresponding to a *d*-spacing of 16 Å, consistent with the average distance between the main chains of PMA-POSS [28]. Therefore, strong aggregation, through crystallization of the POSS nanoparticles, disrupted the intermolecular hydrogen bonding between the C=O units of the PMAPOSS segments and the OH groups of the PVPh segments. When we recorded the XRD patterns of the PVPh60-*co*-PMAPOSS40 random copolymer at various temperatures (Figure 11B), it was revealed that the crystalline peaks from the POSS nanoparticles disappeared when the temperature was 180 °C (i.e., higher than the glass transition temperature), consistent with the DSC analyses in Figure 6. This result is consistent with the POSS domains being disordered or their crystalline size being limited in the PVPh-*co*-PMAPOSS random copolymers at higher temperatures [36].

## 4. Conclusions

We have synthesized PVPh-*co*-PMAPOSS random copolymers through free random copolymerization and subsequent hydrolysis. The well-defined POSS nanoparticles were readily incorporated into the PVPh segments with various concentrations as the POSS feed ratio was varied. We characterized the microstructures, thermal properties, and hydrogen bonding interactions of these random copolymers through NMR spectroscopic, FTIR spectroscopic, GPC, and DSC analyses. Hydrogen bonding interactions were absent in the PVPh/PMAPOSS blend systems due to a strong screening effect. The PVPh-*co*-PMAPOSS random copolymers, however, featured intramolecular hydrogen bonding. Accordingly, the screening effect was minimized in the hydrogen-bonded copolymer system. 

## Figures and Tables

**Table 1:** Effect of MAPOSS feeding ratio on PAS-*co*-PMAPOSS random copolymers.

Nomenclature	Molar Ratio		Weight Ratio		*M*_n_^b^ (g/mol)	PDI^b^	*T*_g_^c^ (°C)
	Feeding	Product	Feeding	Product^a^			
Pure PAS	100/0	100/0	100/0	100/0	17,700	1.61	127
PAS90-*co*-PMAPOSS10	98.13/1.87	97.26/2.74	90/10	85.92/14.08	19,800	1.77	122
PAS80-*co*-PMAPOSS20	95.89/4.11	95.7/4.3	80/20	79.28/20.72	24,300	1.53	117
PAS70-*co*-PMAPOSS30	93.14/6.86	93.82/6.18	70/30	72.29/27.71	27,700	1.48	110
PAS60-*co*-PMAPOSS40	89.72/10.28	91.51/8.49	60/40	64.94/35.06	13,700	1.63	106
PAS50-*co*-PMAPOSS50	85.35/14.65	88.27/11.73	50/50	56.39/43.61	29,500	1.32	105
Pure PMAPOSS	0/100	0/100	0/100	0/100	38,600	1.07	56

^a^ Determined using ^1^H NMR spectroscopy. ^b^ Determined using GPC and a PS standard curve in THF. ^c^ Determined using DSC (heating rate: 20 °C/min).
